# Impact of sputum gross appearance and volume on smear positivity of pulmonary tuberculosis: a prospective cohort study

**DOI:** 10.1186/1471-2334-12-172

**Published:** 2012-08-01

**Authors:** Soon Ho Yoon, Nyoung Keun Lee, Jae Joon Yim

**Affiliations:** 1 Incheon branch, Korean National Tuberculosis Association, 16 Juanyeok-gil, Incheon, South Korea; 2 Department of Radiology, Seoul National University College of Medicine, 28, Yongon-dong, Chongno-gu, Seoul, 110-744, South Korea; 3 Division of Pulmonary and Critical Care Medicine, Department of Internal Medicine and Lung Institute, Seoul National University College of Medicine, 28, Yongon-dong, Chongno-gu, Seoul, 110-744, South Korea; 4 Seoul National University College of Medicine, Chongno-gu, Seoul, 110-744, South Korea

**Keywords:** Smear microscopy, Sputum, Tuberculosis

## Abstract

**Background:**

Although checking specimen quality upon sputum collection for acid-fast smear of suspected tuberculosis (TB) cases is recommended, this procedure is based on expert opinion. The purpose of this study was to elucidate the impact of sputum gross appearance and volume on smear positivity among patients with suspected pulmonary TB, according to sex.

**Methods:**

From November 2010 through June 2011, we enrolled consecutive patients suspected to have active pulmonary TB. The association of sputum gross appearance and volume with smear positivity, along with other variables possibly affecting smear positivity such as symptoms, disease extent, and cavity on chest radiograph, were investigated.

**Results:**

Among 2,439 patients undergoing TB examination, 170 (113 men, 57 women) with active pulmonary TB were enrolled. They submitted 492 sputa. There were 73 smear-positive patients (42.9%) and 164 smear-positive sputa (33.3%). While gross appearance was associated with smear positivity in both sexes (purulent or blood-tinged sputum (rather than mucoid sputum or saliva); odds ratio (OR), 2.05, 95% confidence interval (CI), 1.21–3.47 in men; OR, 2.78, 95% CI, 1.23–6.26 in women), the amount of sputum specimens was associated with smear positivity in only female patients (≥4 ml versus <4 ml; OR, 4.96, 95% CI, 1.98–12.37).

**Conclusions:**

Sputum gross appearance and volume were associated with smear positivity. A volume of 4 ml seems to be the the minimum sputum volume acceptable for smear microscopy in females suspected of TB. Those suspected of TB should be encouraged to expectorate grossly qualified sputum specimens.

## Background

Tuberculosis (TB) remains a major public health problem, and the global burden of TB is enormous. In 2010, 8.8 million cases of TB were reported, with 1.1 million deaths among those who were HIV-negative and an additional 0.35 million deaths from HIV-associated TB [1]. To eradicate TB, early detection and treatment of smear-positive pulmonary TB is a top priority. Smear microscopy is still the most crucial test for the diagnosis of pulmonary TB, especially in countries with limited resources. Accordingly, smear microscopy quality assurance has been emphasized in the WHO Directly Observed Treatment Short Course (DOTS) strategy [2]. Besides smear microscopy quality assurance, the importance of sputum specimen quality has been also suggested. Sputum-submission instruction has been reported to improve detection of smear-positive TB, especially in females suspected of TB [3-6].

However, although such instruction is proved to be useful for optimizing the quality of sputum specimens, at most half of instructed patients submitted adequate sputum specimens [3,7,8]. Considering that WHO plans to change the current “spot-morning” sputum submission to “spot-spot” sputum submission for smear microscopy [9,10] and that the smear positivity yield of spot sputum is lower than that of morning sputum [11], development of quality criteria that could guarantee sputum specimens of adequate quality is crucial. Although various kinds of quality criteria regarding specimen gross appearance and volume [3-6,8,12-14] are recommended to be checked upon collection, these criterias are based not on a well-designed study, but on expert opinion [15]. The purpose of this study was to elucidate the impact of sputum specimen gross appearance and volume on smear positivity in patients suspected to have pulmonary TB, according to sex.

## Methods

### Subjects and sputum collection

From November 15, 2010 through June 30, 2011, we enrolled consecutive persons suspected to have active pulmonary TB at eight primary public health centers in Incheon City, Republic of Korea. Participants were asked to submitted three sputum specimens, consisting of one spot and two subsequent morning sputa. Prior to submission, they were instructed in detail concerning the submission of adequate sputum specimens, including the importance of sputum quality and how to expectorate properly. However, we did not mention a target volume. Persons with a history of previous TB treatment, or who had received TB medication within the past 60 days, or who did not submit two morning sputa, were excluded from the study. The study protocol was approved by the institutional review board of Seoul National University Hospital. Written informed consent was obtained from all participants.

### Sample size calculation

To calculate sample size, we reviewed the results of acid-fast bacilli (AFB) smears between March 1, 2010 and August 30, 2011. Sputum volume was recorded as ≥3 or <3 ml. The difference in smear positivity between saliva and mucoid or purulent specimens was 21.4%, and that between sputum <3 and ≥3 ml was 17.6%. Based on the above findings and allowing 5% for type I and 20% for type II errors, the sample sizes estimated using the generalized estimating equation (GEE) were 82 patients for sputum appearance (correlation coefficient, 0.7186) and 170 for sputum volume (correlation coefficient, 0.7089).

### Collection of demographic and clinical characteristics

Demographic information and clinical characteristics were collected using a questionnaire. The questionnaire included demographic information (age, sex, history of smoking) and clinical characteristics (respiratory symptoms such as cough, sputum, and hemoptysis, as well as general symptoms including fever, night sweats, and weight loss).

### Radiographic evaluation

A posteroanterior chest radiograph was obtained using digital radiography at 110–120 kVp and 2.5–4.0mAs. Each chest radiograph was evaluated for the presence of a cavity and categorized for TB extent into minimal, moderate, and far-advanced, independently by two board-certified radiologists [16]. Any discrepancies were resolved by consensus between the two radiologists during a subsequent review.

### Sputum gross evaluation and volume determination

Two technicians independently evaluated the gross appearance and volume of sputum specimens. The gross appearance of sputum specimens was classified into saliva, mucoid, purulent, or blood-stained sputum [17]. The submitted specimens having clear and watery appearance without any viscosity was designated as saliva. After the exclusion of saliva, the differentiation between mucoid and purulent sputums was based on a five-point sputum color chart (BronkoTest; Heredilab Inc., Salt Lake City, UT, USA). Colors 1 and 2 were regarded as mucoid and colors 3 to 5 as purulent sputum. The sputum specimens having a pink to reddish color besides of color chart was designated as blood-stained sputum. If content of sputum specimens were heterogenous, a predominant portion was used to assess the quality of sputum specimens. Specimen volume was assessed in millimeters by comparing the gradation of a reference container to the volume in the specimen container. Discrepancies were resolved by consensus between the two technicians.

### AFB smear microscopy

Submitted sputa were examined microscopically for the presence of AFB by Ziehl–Nielson staining at each public health center. In addition, specimens were sent to the local reference laboratory center (Korean National Tuberculosis Association, Incheon branch), which was in charge of mycobacterial examinations in Incheon City. In the case of smearing sputum specimens with inhomogenous content, the most purulent portion of sputum specimens was sampled with platinum wire loop. Slides were stained with Auramin-O stain and examined using fluorescence microscopy by the chief technician, who was blinded to the result of Ziehl–Nielson microscopy. The participants were regarded as having smear-positive pulmonary TB if the result of either Ziehl–Nielson or fluorescence microscopy was one or more smears of grade 1+ or higher (10–99 bacilli per 100 fields)(12).

### Mycobacterial culture and TB confirmation

The national reference standard quality-assured culture was performed at the local reference laboratory center. First (spot) and second (morning) sputum specimens underwent solid culture (Ogawa media), and the third (morning) sputum specimen underwent both solid culture (2% Ogawa media, Korean Institute of Tuberculosis, Korea) and liquid culture (BACTEC MGIT (mycobacteria growth indicator tube) 960 culture; BD Microbiology Systems, Sparks, MD, USA). Specimens were decontaminated using 4% NaOH for solid culture, and standard *N*-acetyl- l-cysteine–NaOH (2%) for liquid culture. The chief technician recorded the results of cultures each Tuesday; cultures were designated negative if colonies were absent after 8 weeks of incubation for solid culture. Liquid cultures were observed for 6 weeks. A diagnosis of TB was confirmed if either *Mycobacterium tuberculosis* was isolated in at least one solid or liquid culture, or if clinical-radiological improvements were achieved with anti-TB treatment.

### Statistical analysis

The demographic and clinical characteristics and sputum profiles of enrolled patients are presented as the median with range or mean ± standard deviation, and compared according to smear-positivity using Fisher’s exact, chi-square, and Mann–Whitney tests for categorical variables and Student’s *t*-test for continuous variables. Variables including gross appearance and volume were evaluated to determine their associations with smear positivity using univariate GEEs, with exchangeable working correlations. After testing for multi-collinearity among the variables using variance inflation factors, multivariate models were conducted using combinations of variables that exhibited *P* values <0.05 in the univariate analysis. The multivariate model for predicting smear positivity was judged to be optimized when the quasi-likelihood under the independence model criterion in GEE analysis showed the lowest value. The association of sputum volume with smear positivity was investigated after stratification according to sex not only in all enrolled TB cases but also in culture confirmed TB cases. In addition, the association between disease severity/smoking and volume of submitted sputum was analyzed in both sexes to evaluate the impact of possible confounders affecting the association between volume of sputum specimens and smear positivity.

Inter-observer agreement regarding smear positivity between public health centers and the local laboratory center, radiographic evaluation between radiologists, and sputum gross appearance and volume between technicians was assessed with the κ statistic [18]. A κ value <0.20 indicated poor agreement; a κ value of 0.21–0.40, fair agreement; a κ value of 0.41–0.60, moderate agreement; a κ value of 0.61–0.80, good agreement; and a κ value of more than 0.81, excellent agreement.

Data were analyzed using the SPSS software package (ver. 17.0; SPSS, Inc., Chicago, IL, USA). A two-sided significance level of 5% was used for all analyses.

## Results

### Demographic and clinical characteristics

**Figure 1:**
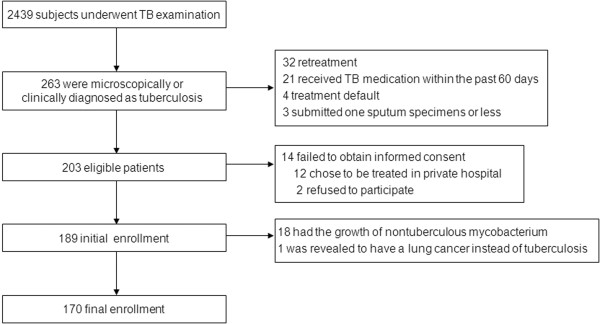
Flow chart of the study group enrollment process.

Among 2,439 patients undergoing a TB examination in public health centers, a total of 263 patients were microscopically or clinically diagnosed with pulmonary TB. Of these, 74 patients who met the exclusion criteria or who did not provide informed consent were excluded. In addition, 19 patients diagnosed with nontuberculous mycobacterial lung diseases or lung cancer were excluded. Finally, 170 with pulmonary TB were included and analyzed (Figure 1). Of these, 101 (59.4%) had culture-positive TB and 69 (40.6%), clinically diagnosed TB. One-hundred and thirteen (66.4%) were male. Their median age was 44.0 years (range, 16–87 years). Ninety-five (55.8%) patients were ever-smokers. A total of 113 (66.4%) had one or more respiratory symptoms, most commonly, cough and sputum. One-hundred one patients (58.8%) had a minimal TB extent on their chest radiographs; 50 (29.4%) had a moderate extent, and 19 (11.2%) had a severe extent. In addition, 41 (24.1%) had cavitary lesions (Table 1).

**Table 1:** Demographic, clinical and radiographic characteristics of the enrolled patients

	**All participant (N = 170)**	**Male TB patient (N = 113)**	**Female TB patient (N = 57)**	**P-value**
**Age**	43.1 ± 19.1	42.7 ± 18.2	44.1 ± 20.7	0.646
**Body mass index**	21.1 ± 2.8	19.3 ± 6.2	17.3 ± 9.0	0.148
**Respiratory symptoms**				
any symptom	66.4% (113/170)	69.0% (78/113)	61.4% (35/57)	0.390
cough	55.9% (95/170)	58.4% (66/113)	50.9% (29/57)	0.414
sputum	40.6% (69/170)	44.2% (50/113)	33.3% (19/57)	0.189
hemoptysis	4.1% (7/170)	3.5% (4/113)	5.3% (3/57)	0.688
duration (weeks)	6.1 ± 14.0	5.3 ± 9.2	6.8 ± 19.8	0.499
**General symptoms**	30.6% (52/170)	34.5% (39/113)	22.8% (13/57)	0.081
**Smoking**				
Current Smoker	32.9% (56/170)	45.1% (51/113)	8.8% (5/57)	<0.001
Ex-smoker	22.9% (39/170)	24.8% (28/113)	19.3% (11/57)	0.448
Pack year	10.9 ± 15.6	13.5 ± 15.7	3.4 ± 12.0	<0.001
**Radiographic extent**				
minimal	59.4% (101/170)	51.3% (58/113)	75.4% (43/57)	0.009
moderate	29.4% (50/170)	34.5% (39/113)	19.3% (11/57)	
far-advanced	11.2% (19/170)	14.2% (16/113)	5.3% (3/57)	
**Cavity on chest radiograph**	24.1% (41/170)	31.0% (35/113)	10.5% (6/57)	0.004
**Smear positivity**	42.9% (73/170)	46.0% (52/113)	36.8% (21/57)	0.325
**Culture positivity**	59.4% (101/170)	62.8% (71/113)	52.6% (30/57)	0.247

Note. ─ Numbers in parenthesis indicate the number of patients. *TB* tuberculosis.

AFB was observed in one or more sputum smears from 73 (42.9%) patients (smear-positive TB), including 52 (46.0%) men and 21 (36.8%) women. Sputum culture revealed *M*. *tuberculosis* in 101 (59.4%) patients, including 71 men (62.8%) and 30 women (52.6%). Current smokers were more common among male than female patients with TB (45.1% vs. 8.8%; *P* < 0.001). Extent of TB was wider ( *P* = 0.009) and cavities were more common ( *P* = 0.004) in men.

### Sputum gross appearance and volume

A total of 170 patients submitted 492 sputa. Most (152, 89.4%) submitted one spot and two subsequent morning sputa. Mucoid (42.3%) and purulent (33.3%) sputa were common, and the median sputum volume was 3.0 ml (range, 1.0–8.0 ml). The distribution of gross appearance of sputum did not differ by sex, while women submitted significantly lower volumes of sputum than men (median 2 ml vs. 3 ml; *P* = 0.001)(Table 2).

**Table 2:** Sputum profiles of the enrolled patients

	**All sputa (N = 492)**	**Sputa from males (N = 329)**	**Sputa from females (N = 163)**	**P-value**
**Sputum order**				0.551
spot	36.1% (178/492)	35.3% (116/329)	38.0% (62/163)	
morning	63.9% (314/492)	64.7% (213/329)	62.0% (101/163)	
**Sputum order**				0.961
1^st^	34.5% (170/492)	34.3% (113/329)	35.0% (57/163)	
2^nd^	34.5% (170/492)	34.3% (113/329)	35.0% (57/163)	
3^rd^	31.0% (152/492)	31.4% (103/329)	30.0% (49/163)	
**Gross appearance**				0.129
saliva	22.0% (108/492)	19.5% (64/329)	27.0% (44/163)	
mucoid	42.3% (208/492)	41.6% (137/329)	43.6% (71/163)	
purulent	33.3% (164/492)	36.2% (119/329)	27.6% (45/163)	
blood-stained	2.4% (12/492)	2.7% (9/329)	1.8% (3/163)	
**Volume**				0.001
<1 ml	22.0% (108/492)	17.6% (58/329)	30.7% (50/163)	
1 ml ≤ <2 ml	27.6% (136/492)	27.1% (89/329)	28.8% (47/163)	
2 ml ≤ <3 ml	25.6% (126/492)	27.4% (90/329)	22.1% (36/163)	
3 ml ≤ <4 ml	14.0% (69/492)	17.0% (56/329)	8.0% (13/163)	
4 ml ≤ <5 ml	7.3% (36/492)	7.9% (26/329)	6.1% (10/163)	
≥ 5 ml	3.5% (17/492)	3.0% (10/329)	4.3% (7/163)	

Note. ─ Numbers in parenthesis indicate the number of sputa.

### Kappa statistics

The kappa values were 0.75 for smear positivity, 0.57 for disease extent, 0.64 for the presence of cavity on chest radiograph, and 0.69 and 0.67 for gross appearance and volume, respectively.

### Predictors of smear positivity in male and female patients with TB

Predictors of smear positivity differed by sex (Tables 3, 4 and 5). In male patients, the final model predicting smear positivity included age, presence of hemoptysis, duration of symptoms (≥3 weeks), presence of general symptoms, presence of a cavity, and sputum gross appearance. However, sputum volume was not included. Among them, duration of respiratory symptoms (odds ratio (OR), 3.17, 95% confidence interval (CI), 1.29–7.77), presence of a cavity on chest radiograph (OR, 3.50, 95% CI, 1.54–7.95), and sputum gross appearance (purulent or blood-tinged sputum, rather than mucoid or saliva; OR, 2.05, 95% CI, 1.21–3.47), showed significant associations with smear positivity.

**Table 3:** Results of a univariate Generalized Estimating Equation Model Predicting smear positivity according to sex

	**Male**		**Female**	
	**Odd ratio (95% Cl)**	**P-value**	**Odd ratio (95% Cl)**	**P-value**
**Age**				
40-59 years (versus < 40 years)	2.76 (1.24-6.14)	0.013	1.11 (0.32-3.89)	0.867
≥ 60 years (versus < 40 years)	1.03 (0.37-2.82)	0.961	2.45 (0.73-8.21)	0.146
**BMI**				
≥23 (versus <23)	1.70 (0.71-4.10)	0.235	1.16 (0.39-3.42)	0.787
**Respiratory symptoms**				
any symptom	3.73 (1.57-8.87)	0.003	3.85 (1.44-10.28)	0.007
cough	3.02 (1.42-6.42)	0.004	5.31 (2.03-13.92)	0.001
sputum	3.72 (1.78-7.77)	<0.001	1.61 (0.56-4.63)	0.376
hemoptysis	2.26 (0.38-13.33)	0.369	7.96 (1.91-33.21)	0.004
duration (<3 weeks versus ≥3 weeks)	3.71 (1.77-7.78)	0.001	4.93 (1.83-13.30)	0.002
**General symptoms**	2.52 (1.19-5.37)	0.016	1.74 (0.44-6.94)	0.431
**Smoking**				
current smoking	1.38 (0.68-2.81)	0.374	0.53 (0.14-2.09)	0.374
previous smoking	1.37 (0.63-2.98)	0.430	0.58 (0.14-2.40)	0.451
1-10 pack-years (versus 0 pack-years )	1.85 (0.65-5.25)	0.250	0.58 (0.14-2,49)	0.464
≥ 10 pack-years (versus 0 pack-years )	2.93 (1.22-7.07)	0.016	0.65 (0.18-2.36)	0.507
**Radiographic extent**				
moderate or far-advanced (versus minimal)	4.42 (2.07-9.45)	<0.001	1.23 (0.37-4.10)	0.732
cavity	5.27 (2.24-11.47)	<0.001	2.02 (0.342-11.91)	0.438
**Gross appearance of sputum specimens**				
sputum (versus saliva )	1.70 (1.08-2.67)	0.021	1.51 (0.70-3.29)	0.297
purulent, bloody (versus saliva, mucoid)	2.04 (1.33-3.12)	0.001	2.96 (1.46-6.01)	0.003
**Volume of sputum specimens**				
≥1 ml (<1 ml versus ≥1 ml)	1.18 (0.74-1.88)	0.482	2.81 (1.43-5.51)	0.003
≥2 ml (<2 ml versus ≥2 ml)	1.10 (0.87-1.39)	0.433	2.27 (0.99-5.17)	0.052
≥3 ml (<3 ml versus ≥3 ml)	1.12 (0.87-1.44)	0.396	1.74 (0.57-5.31)	0.331
≥4 ml (<4 ml versus ≥4 ml)	1.09 (0.90-1.32)	0.372	3.89 (1.89-7.98)	<0.001
≥5 ml (<5 ml versus ≥5 ml)	1.01 (0.87-1.16)	0.933	3.31 (0.84-13.06)	0.088

Note. ─ *CI* confidence interval.

**Table 4:** Results of a multivariate Generalized Estimating Equation Model Predicting smear positivity in male TB patients

	**Estimate**	**Total**	
		**Odd ratio (95% Cl)**	**P-value**
**(Intercept)**	−2.065		
**Age**			
40-59 years (versus < 40 years)	0.104	1.10 (0.44-2.78)	0.825
≥ 60 years (versus < 40 years)	0.479	2.09 (0.47-5.52)	0.181
**Respiratory symptoms**			
hemoptysis	0.981	2.67 (0.31-22.69)	0.369
duration (<3 weeks versus ≥3 weeks)	1.152	3.17 (1.29 -7.77)	0.012
**General symptoms**	0.796	2.21 (0.94-5.25)	0.070
**Radiographic extent**			
cavity	1.253	3.50 (1.54-7.95)	0.003
**Gross appearance of sputum specimens**			
purulent, bloody (versus saliva, mucoid)	0.718	2.05 (1.21-3.47)	0.007

Note. ─ *CI* confidence interval.

**Table 5:** Results of a multivariate Generalized Estimating Equation Model Predicting smear positivity in female TB patients

	**Estimate**	**Total**	
		**Odd ratio (95% Cl)**	**P-value**
**(Intercept)**	−2.705		
**Respiratory symptoms**			
hemoptysis	1.314	3.72 (0.76-18.19)	0.105
duration (<3 weeks versus ≥3 weeks)	1.575	4.83 (1.71-13.64)	0.003
**General symptoms**	0.638	1.89 (0.40-9.01)	0.424
purulent, bloody (versus saliva, mucoid)	1.021	2.78 (1.23-6.26)	0.014
**Volume of sputum specimens**			
≥4 ml (<4 ml versus ≥4 ml)	1.600	4.96 (1.98-12.37)	0.001

Note. ─ *CI* confidence interval.

In women, the final model included the presence of hemoptysis, duration of symptoms (≥3 weeks), presence of general symptoms, gross appearance, and the volume of sputum specimen. Among them, duration of respiratory symptoms (OR, 4.83, 95% CI, 1.71–13.64), gross appearance (purulent or blood-tinged sputum, rather than mucoid or saliva; OR, 2.78, 95% CI, 1.23–6.26), and sputum volume (≥4 ml vs. <4 ml; OR, 4.96, 95% CI, 1.98–12.37) were significantly associated with smear positivity in female patients. The results of univariate and multivariate GEE analyses in all enrolled patients regardless of sex are shown in the Additional file 1: Appendix A-B.

Among the culture confirmed cases (n = 101; 71 male patients and 30 female patients), BMI (≥23 vs. <23; OR, 5.58, 95% CI, 1.51-20.64) and gross appearance of sputum (purulent or blood-tinged sputum, rather than mucoid or saliva; OR, 6.84, 95% CI, 3.12-15.01) were significantly associated with smear positivity in male patients on multivariate analyses. In female patients, duration of respiratory symptoms (OR, 8.91, 95% CI, 2.76–28.81) and sputum volume (≥4 ml vs. <4 ml; OR, 7.99, 95% CI, 2.13–30.07) were significantly associated with smear positivity. (Refer to Additional file 1: Appendix C-E).

### Impact of disease severity and smoking on volume of sputum specimens in female patients with TB

In male patients, the presence of cavity (OR, 1.45, 95% CI, 0.99–2.14) and amount of smoking (≥ 10 pack-years vs. none; OR, 1.58, 95% CI, 1.02–2.46) were associated with increased volume of submitted sputum. On contrary, neither presence of cavity, nor extent of disease, nor amount of smoking were associated with volume of submitted sputum in females.

## Discussion

Through this prospective study, we showed that both gross appearance and volume of sputum were associated with smear positivity among female patients with pulmonary TB. However, among male patients, only gross appearance was a predictor of smear positivity.

The upgraded gross appearance of submitted sputum in those suspected of TB was proposed to contribute to the increase in smear positivity [3,4,6]. In addition, the number of bacilli on smear positivity can be underestimated in macroscopic poor-quality specimens [3,17,19]. Our study confirmed the importance of sputum gross appearance. Nonetheless, even saliva-like sputum specimens cannot be discarded because they could yield AFB [20-22]. In our study, the AFB positivity of saliva was not trivial (17.6%, 19 of 108).

Sputum volume (≥4 ml) was associated with smear positivity in women, but not in men. Since women are less proficient at expectoration than men [23,24], the chance of expectorating proper sputum during attempts to spit can increase in women with increasing requisite sputum volume. The lack of association between diseases severity/smoking and volume of sputum specimens in females reflects their inferiority at expectoration of sputum and reinforces the importance of volume of submitted sputum. In contrast, the chance of expectorating proper sputum may be relatively constant in men, regardless of the requisite volume, since they can more easily expectorate proper sputum. This possibility is further reinforced by the smear positivity trend being stagnant in men but increasing in women with increasing sputum volume (data not shown). In fact, our result is in accordance with a previous report that smear positivity was increased substantially by provision of brief instructions, but only in females [4]. If available, sputum induction or bronchoscopy can be used as an useful technique to assess smear positivity in TB patients who are unable to submit adequate sputum specimens [25].

Although setting a minimum volume of required sputum for those suspected of TB has been reported to be effective, no consensus exists as to how much is adequate. Collection of 2, 3, or 5 ml of sputum has been recommended, or tested as a surrogate for an adequate sample [3-5,8,12,13], without detailed analysis. We tested various volumes (1, 2, 3, 4, and 5 ml) to determine the most appropriate minimum volume. Four milliliters was the optimum minimal volume in female suspects. Sputum volume was not associated with smear positivity in a previous study, when the mean specimen volume was 4.0 ml or more [26].

Clinical and radiographic features were associated with smear positivity. First, duration of respiratory symptoms (>3 weeks) was associated with smear positivity in both sexes. Longer symptom duration may reflect TB diagnosis at an advanced stage and so a higher bacilli load, leading to a positive AFB smear [27,28]. However, in this study, the presence of a cavity, a hallmark of high TB burden [29], was associated with smear positivity only in men. We are at present unable to explain why the presence of a cavity was not associated with smear positivity among women. The relative rarity of cavities in females (10.5%) may have hindered the proper evaluation of their association with smear positivity.

## Conclusions

Sputum specimen quality and volume were associated with smear positivity. A volume of 4 ml was the optimum minimum sputum volume acceptable for smear microscopy in women suspected of having TB. Those suspects of both sexes should be encouraged to expectorate grossly qualified sputum specimens.

## Competing interests

The authors declare that they have no competing interest.

## Authors’ contribution

SHY was responsible for study design, patient collection, data collection, statistical analysis, data interpretation, drafting and writing the manuscript, NKL contributed to data collection, and drafting the manuscript, JJY was responsible for study design, data interpretation, drafting and writing the manuscript. All authors read and approved the final manuscript.

## Pre-publication history

The pre-publication history for this paper can be accessed here:

http://www.biomedcentral.com/1471-2334/12/172/prepub

## Supplementary Material

Additional file 1 Appendix Click here for file
