# Supplementary material for: Impact of sputum gross appearance and volume on smear positivity of pulmonary tuberculosis: a prospective cohort study
Source: BMC Infect Dis. 2012 Aug 1;12:172. doi: 10.1186/1471-2334-12-172 (PMC3449203; doi:10.1186/1471-2334-12-172)
Supplement: Additional file 1 — Appendix [file 1471-2334-12-172-S1.docx]

Appendix

Appendix A.

**Results of a univariate Generalized Estimating Equation Model Predicting smear positivity in all enrolled patients**

|  | **Total** | |
| --- | --- | --- |
|  | **Odd Ratio** | **P-value** |
|  | **(95% Cl)** |  |
| **Age** |  |  |
| 40-59 years (versus < 40 years) | 2.33 (1.20-4.49) | 0.012 |
| ≥ 60 years (versus < 40 years) | 1.31 (0.60-2.84) | 0.500 |
| **Sex** |  |  |
| male | 2.34 (1.29-4.43) | 0.006 |
| **Body mass index** |  |  |
| ≥23 (versus <23) | 1.25 (0.64-2.42) | 0.513 |
| **Respiratory symptoms** |  |  |
| any symptom | 3.84 (1.93-7.62) | <0.001 |
| cough | 3.52 (1.90-6.53) | <0.001 |
| sputum | 3.11 (1.71-5.63) | <0.001 |
| hemoptysis | 3.44 (1.04-11.03) | 0.044 |
| duration (<3 weeks versus ≥3weeks) | 4.08 (2.23-7.46) | <0.001 |
| **General symptoms** | 1.95 (1.04-3.65) | 0.036 |
| **Smoking** |  |  |
| current smoking | 1.63 (0.89-3.03) | 0.115 |
| previous smoking | 1.21 (0.62-2.34) | 0.579 |
| 1-10 pack-years (versus 0 pack-years ) | 1.26 (0.57-2.77) | 0.569 |
| ≥ 10 pack-years (versus 0 pack-years ) | 2.74 (1.42-5.31) | 0.003 |
| **Radiographic extent** |  |  |
| moderate or far-advanced (versus minimal) | 3.56 (1.96-6.48) | <0.001 |
| cavity | 5.07 (2.56-10.00) | <0.001 |
| **Gross appearance of sputum specimens** |  |  |
| sputum (versus saliva ) | 1.69 (1.15-2.49) | 0.007 |
| purulent, bloody (versus saliva, mucoid) | 2.26 (1.58-3.23) | <0.001 |
| **Volume of sputum specimens** |  |  |
| ≥1ml (<1ml versus ≥1ml) | 1.44 (1.02-2.03) | 0.039 |
| ≥2ml (<2ml versus ≥2ml) | 1.30 (0.97-1.74) | 0.080 |
| ≥3ml (<3ml versus ≥3ml) | 1.15 (0.84-1.58) | 0.387 |
| ≥4ml (<4ml versus ≥4ml) | 1.44 (1.08-1.92) | 0.014 |
| ≥5ml (<5ml versus ≥5ml) | 1.39 (0.76-2.53) | 0.287 |

Note. ─ CI = confidence interval.

Appendix B.

**Results of a multivariate Generalized Estimating Equation Model Predicting smear positivity in all enrolled patients**

|  |  | **Total** | |
| --- | --- | --- | --- |
|  | **Estimate** | **Odd Ratio** | **P-value** |
|  |  | **(95% Cl)** |  |
| **(Intercept)** | -2.684 |  |  |
| **Age** |  |  |  |
| 40-59 years (versus < 40 years) | 0.327 | 1.39 (0.68-2.85) | 0.373 |
| ≥ 60 years (versus < 40 years) | 0.025 | 1.03 (0.44-2.41) | 0.953 |
| **Sex** |  |  |  |
| male | 0.522 | 1.69 (0.88-3.24) | 0.117 |
| **Respiratory symptoms** |  |  |  |
| hemoptysis | 1.490 | 4.44 (1.09-18.08) | 0.038 |
| duration (<3 weeks versus ≥3weeks) | 1.072 | 2.92 (1.52-5.60) | 0.001 |
| **General symptoms** | 0.574 | 1.78 (0.87-3.64) | 0.116 |
| **Radiographic extent** |  |  |  |
| cavity | 1.128 | 3.09 (1.50-6.37) | 0.002 |
| **Gross appearance of sputum specimens** |  |  |  |
| purulent, bloody (versus saliva, mucoid) | 0.848 | 2.34 (1.53-3.57) | <0.001 |
| **Volume of sputum specimens** |  |  |  |
| ≥4ml (<4ml versus ≥4ml) | 0.335 | 1.40 (0.89-2.19) | 0.141 |

Note. ─ CI = confidence interval.

Appendix C.

**Results of a univariate Generalized Estimating Equation Model Predicting smear positivity according to sex in culture confirmed TB patients**

|  | **Male** | | **Female** | |
| --- | --- | --- | --- | --- |
|  | **Odd Ratio** | **P-value** | **Odd Ratio** | **P-value** |
|  | **(95% Cl)** |  | **(95% Cl)** |  |
| **Age** |  |  |  |  |
| 40-59 years (versus < 40 years) | 2.41 (0.88-6.58) | 0.087 | 1.12 (0.28-4.53) | 0.879 |
| ≥ 60 years (versus < 40 years) | 1.57 (0.50-4.88) | 0.436 | 1.83 (0.45-7.34) | 0.397 |
| **BMI** |  |  |  |  |
| ≥23 (versus <23) | 4.30 (1.22-15.20) | 0.024 | 0.91 (0.23-3.57) | 0.886 |
| **Respiratory symptoms** |  |  |  |  |
| any symptom | 1.34 (0.44-4.07) | 0.604 | 3.75 (1.31-10.73) | 0.014 |
| cough | 1.83 (0.74-4.51) | 0.192 | 4.66 (1.60-13.52) | 0.005 |
| sputum | 2.41 (1.01-5.77) | 0.048 | 1.75 (0.53-5.86) | 0.361 |
| hemoptysis | 2.27 (0.28-18.58) | 0.446 | 1.96 (0.65-5.95) | 0.234 |
| duration (<3 weeks versus ≥3weeks) | 1.51 (0.62-3.64) | 0.362 | 6.67 (2.32-19.16) | <0.001 |
| **General symptoms** | 2.07 (0.82-5.18) | 0.122 | 1.67 (0.40-6.99) | 0.485 |
| **Smoking** |  |  |  |  |
| current smoking | 1.26 (0.53-2.99) | 0.609 | 3.04 (0.81-11.49) | 0.101 |
| previous smoking | 1.05 (0.43-2.56) | 0.920 | 1.24 (0.30-5.09) | 0.761 |
| 1-10 pack-years (versus 0 pack-years ) | 1.11 (0.32-3.89) | 0.870 | 2.11 (0.49-9.07) | 0.474 |
| ≥ 10 pack-years (versus 0 pack-years ) | 1.61 (0.55-4.70) | 0.381 | 1.95 (0.67-5.61) | 0.514 |
| **Radiographic extent** |  |  |  |  |
| moderate or far-advanced (versus minimal) | 1.56 (0.62-3.96) | 0.346 | 1.50 (0.43-5.58) | 0.545 |
| cavity | 1.94 (0.81-4.62) | 0.136 | 2.08 (0.27-16.05) | 0.481 |
| **Gross appearance of sputum specimens** |  |  |  |  |
| sputum (versus saliva ) | 5.29 (2.11-13.30) | <0.001 | 1.60 (0.59-4.32) | 0.354 |
| purulent, bloody (versus saliva, mucoid) | 6.53 (3.12-13.70) | <0.001 | 2.23 (0.93-5.34) | 0.073 |
| **Volume of sputum specimens** |  |  |  |  |
| ≥1ml (<1ml versus ≥1ml) | 1.48 (0.56-3.88) | 0.430 | 3.60 (1.04-12.44) | 0.043 |
| ≥2ml (<2ml versus ≥2ml) | 1.40 (0.75-2.59) | 0.292 | 2.14 (0.80-5.71) | 0.128 |
| ≥3ml (<3ml versus ≥3ml) | 1.53 (0.78-2.97) | 0.215 | 1.29 (0.40-4.19) | 0.676 |
| ≥4ml (<4ml versus ≥4ml) | 1.20 (0.47-3.07) | 0.703 | 4.73 (1.65-13.58) | 0.004 |
| ≥5ml (<5ml versus ≥5ml) | 1.26 (0.24-6.73) | 0.784 | 3.00 (0.67-13.40) | 0.150 |

Note. ─ CI = confidence interval.

Appendix D.

**Results of a multivariate Generalized Estimating Equation Model Predicting smear positivity in culture confirmed male TB patients**

|  |  | **Total** | |
| --- | --- | --- | --- |
|  | **Estimate** | **Odd Ratio** | **P-value** |
|  |  | **(95% Cl)** |  |
| **(Intercept)** | -1.007 |  |  |
| **Age** |  |  |  |
| 40-59 years (versus < 40 years) | 0.705 | 2.03 (0.66-6.19) | 0.216 |
| ≥ 60 years (versus < 40 years) | 0.644 | 1.90 (0.61-5.97) | 0.269 |
| **BMI** |  |  |  |
| ≥23 (versus <23) | 1.720 | 5.58 (1.51-20.64) | 0.010 |
| **Respiratory symptoms** |  |  |  |
| sputum | 0.503 | 1.65 (0.64 -4.30) | 0.302 |
| **Gross appearance of sputum specimens** |  |  |  |
| purulent, bloody (versus saliva, mucoid) | 1.923 | 6.84 (3.12-15.01) | <0.001 |

Note. ─ CI = confidence interval.

Appendix E.

**Results of a multivariate Generalized Estimating Equation Model Predicting smear positivity in culture confirmed female TB patients**

|  |  | **Total** | |
| --- | --- | --- | --- |
|  | **Estimate** | **Odd Ratio** | **P-value** |
|  |  | **(95% Cl)** |  |
| **(Intercept)** | -2.268 |  |  |
| **Respiratory symptoms** |  |  |  |
| duration (<3 weeks versus ≥3weeks) | 2.188 | 8.91 (2.76-28.81) | <0.001 |
| **Volume of sputum specimens** |  |  |  |
| ≥4ml (<4ml versus ≥4ml) | 2.078 | 7.99 (2.12-30.07) | 0.002 |

Note. ─ CI = confidence interval.
